# Supplementary material for: Cryolipolysis-induced abdominal fat change: Split-body trials
Source: PLoS One. 2020 Dec 29;15(12):e0242782. doi: 10.1371/journal.pone.0242782 (PMC7771684; doi:10.1371/journal.pone.0242782)
Supplement: S1 File — (PDF) [file pone.0242782.s002.pdf]

Research Proposal

1. Title

Cryolipolysis induced abdominal fat change

2. Principal investigator, Co-investigator & Coordinator

Kyu Rae Lee (P I), I.C. Hwang, K.K. Kim (Co investigator); SK Kim, HS Moon (Coordinator)

3. Manager for Medical Device

Kyu Rae Lee (P. I.)

4. Funding and institution

Gachon University Fund, Gachon University

5. Other co worked institution

Single institutional study

6. Place & predicted duration for study

Gachon university Dong Incheon Gil Hospital, 12weeks

7. Inclusion Criteria

Apparently healthy subjects

8. Background

Since 1997, Epstein found that superficial fat tissue would be disappeared through cold induced apotosis during his study for panniculitis, Meinstein and Anderson (photodermatology, Harvard) investigated to apply this principle for cosmetic and lipo-sculpture purpose. US FDA approved cryolipolysis in 2010. It was reported (2004, NEJM) that large volume abdominal liposuction did not significantly improve obesity-associated metabolic abnormalities. Furthermore, it is invasive method with risks of infection, hemorrhage, scarring, therefore is not recommendable. Plenty human clinical trial about abdominal fat reduction had been performed with Zeltiq since FDA approval, investigator (2017) published the change of abdominal fat by single cryolipolysis with cryo-Elsa as pilot study in single group without placebo.

9. Purpose and Hypothesis

Because it is very difficult to match the placebo group according to same body composition, we compared the difference of cross sectional areas of adipose tissues of both areas after single session cryolipolysis on left side abdomen (case) only for 60 minutes (split body clinical trial).
BMI, waist circumferences, and photographs, body fat percent were compared after fasting over 8 hours at initial, 6th week, and 12th week visit. In addition, the food intake and physical activity were monitored and calculated in Kcal by 4-day food diary at initial, 6th week, and 12th week visit. The cross sectional areas of both abdominal adipose tissues (VAT, SAT) were compared at initial and 12th week visit. We hypothesized that single cryolipolysis would reduce the cross sectional areas of left side visceral adipose tissue (VAT) than those of right VAT.

10. Sample size and its rationale

The minimum required subjects were 16 in order to compare the differences of mean (cross sectional areas) of single population according to G power program (alpha=0.05, beta>80%). More than 1.5 times of them, 25 subjects were considered as initial recruited target participants. SPSS windows package (Version 18, IL, USA) would be performed for statistical analysis.

11. Inclusion/Exclusion Criteria

Included were apparently healthy men or women with visible fat on the abdomen with BMI over 22 or those without change over 5% of the former weight within the past 6 months.
Excluded were those who had a known history of cryoglobulinemia, cold urticaria, impaired peripheral circulation, Raynauds disease or paroxysmal cold hemogobulinemia. Individuals who had metabolic derangement such as Type 2 DM, dyslipidemia or any medications to regulate weight or fat mass over 6 months were exempt. In addition, excluded were pregnant or lactating women within 6 months or next 3 months.

12. Recruitment method and consent process

Participants will be recruited through Intranet university site, ward bulletin, or outdoor flyers
The present investigators, its associates and institution would not exclude the eligible subjects for a race or socioeconomic status. All best will be provided to enable the eligible subjects partake in the present study and will inform them the purpose. The investigator will explain the enrolled subjects understandable to the study in the

dependent place and make effort them with spontaneous participation.

13. Additional protective protocol for weak risk population

Employees in university hospital have to be interviewed without his/her supervisor in case of recruitment or consent process. He/she could make a decision not to join, which would not influence their employment or job assessment.

Pregnant women or infant or neonates were excluded and university students might not join the study, which would not any influence on their educational process such as mark or class.

14. Design and method

1) Process

① Measurement- Pre and post cryolipolysis; body fat, height, and weight, body fat percent

② Total visit- 4 times- Instruction, 0, 6, 12 week

③single treatment- Cryolipolysis on left side abdomen only for 60 minutes

④Height, weight, and body fat, visual assessment were done after fasting over 8 hours at 0, 6th, and 12th week.

⑤ Frontal, both lateral, and back abdomen were photographed without face and low extremities.

⑥ The cross sectional areas of adipose tissues (VAT, SAT) were measured by CT at 0 and 12th week.

⑦Food intakes were and monitored by software (CanPro. program) through 4-day food diary.

⑧ Adverse event and any complaints were monitored at follow up visit.

2) Blinding will not apply to this study because right side abdomen as placebo is the part of subject's body.

3) Research Schedule:

Itinerary / Item	Screening		Visit	
		Visit 1<br>0 week	Visit 2<br>6 weeek	Visit 3<br>12week
Consent form	O	X	X	X
Inclusion/Exclusion	O	X	X	X
Basic Information	O	X	X	X
Past Medical History	O	X	X	X
Vital Sign	X	O	O	O
Body fat Percent	X	O	X	O
Photograph	X	O	O	O
Cryolipolysis	X	O	X	X
Food/Exercise	X	O	O	O
Adverse Events	X	O	O	O
C-T	X	O	X	O
Satisfaction Score		O		O
Follow up	X	O	O	O

15. Conventional treatment method

Surgical procedure such as lipectomy or liposuction is considered as an effective modality to reduce abdominal fat.

16. benefit and risk for subjects

1) Minor and transient dermatologic lesion might be found after procedure.

2) Pain would be noted; even cold burn might be accompanied in severe case.

3) The participants would get a cosmetic merit for body contour.

17. Assessment for effectiveness and its method

Compare the cross sectional areas of adipose tissue such as VAT, SAT by CT

18. Statistical method

Wilcoxon signed-rank test to assess the difference of waist circumferences, the areas of visceral and subcutaneous adipose tissue, and percent of body fat, visceral to subcutaneous fat ratio.

19. Drop out Criteria

1) Not to agree to attribute

2) Impossible to keep the study duet to the significant adverse event or sudden accident

3) those who does not visit or violation against the guideline or protocol rule

4) hard to keep the continuous investigation for the emergence of severe complication

5) Wrong enrolled illegible subject

6) In Case of inclusion of excluded subjects in the study

7) Those who prevents investigation from carrying the study

20. Intervention Medical Device

1) Rank of Machine 1st degree rank/B-type

2) Model Name CryoEVO/ CryoElsa

3) Mode of action Fat reduction by cooling induced superficial fat

4) Warning for maintenance keep at room temperature, Avoid against high temperature&, humidity

5) Responsibility for management Gachon University, Dong-Incheon Gil Hospital

6) Place for Maintenance Gachon University, Dong-Incheon Gil Hospital

7) Manufacturer Lutronic/Huons (Korea)

21. Anticipated complication and Warning for Use

Please notify to investigator and its associates and visit the hospital if any, because a few of complication such as cold burn after cryolipolysis has been reported.

22. Safety and its report

Definition for AE (Adverse Event)
All of undesirable outcome among the suspicious unexpected results occurring or occurred from the usage of the medical device
SAEs (Serious Adverse Events) are defined as below.
(1) death or fatal risk to life occur
(2) Need to admit or extend to hospital stay
(3) Permanent or severe sequel or functional damage
(4) Congenital malformation or aberration to feus

Record of AE and SAEs

Principal investigator should write AE and SAEs in the record sheet and report to IRB immediately all information including description of AE, occurring date, expired date, strength, relationship with medical device, outcome, treatment, SAEs.
Treatment of AE and SAEs
Those who experienced AE should be monitored fully as possible until their symptoms would be disappeared and abnormal laboratory values would be normalized. In addition, all observed results from cases with adverse events should be written in the case records and patient medical record charts.
Report of SAEs
If any rare and unexpected SAEs happen, investigators and its associates should notify Gachon University IRB and Korea FDA in 7 days. Investigators and its associates would send follow up records including new information SAEs Gachon University IRB and Korea FDA, if need.

23. Compensation protocol for participants

1) The medical service will be done according to the symptom of subject in needed and

its cost has to be covered by principal investigator in case of medical injury related with clinical trial.

2) Investigator gives cultural certificate card (15 US dollar) without condition at each visit, should cover the medical service and its cost to treat drop out subjects who injured related with the clinical trial.

3) Participant may charge the other costs which be not related with the clinical trial

24. Protection for privacy and keeping personal record in secret

All records should be written in anonymous naming and identification number and kept in corded files in computer, all document records have to be kept in the locked office, therefore they should be strictly carried in secret and protected, all of the results is recorded in medical files and Korea FDA review to assess its clinical efficacy and effectiveness.

25. Plan for mid-term analysis including early termination

No plan

26. Data and Safety Monitoring Plan (DSMP)

Investigator and associates will monitor adverse event and specific adverse event and review and follow up periodically on writing the case records and will notify if any. Principal would continue or withdraw the clinical trial according to research protocol.

27. Reference

1. M.G. Berry, Dai Davies, Liposuction: A review of principles and techniques 1748-6815 222 2010 J Plast, Reconstr Aesthet Surg (2010) 64,985-992.
2. Elisa Bellini, Michele P. Grieco, Edoardo Raposio A journey through liposuction and 224 liposculture: Review. Annals of Medicine and Surgery 24 (2017) 53.60.
3. Klein S, Fontana L, Young VL, et al. Absence of an Effect of Liposuction on Insulin 226 Action and Risk Factors for Coronary Heart Disease N Engl J Med 2004;350: 2549.57.
4. M.M. Avram and R.S. Harry, i°Cryolipolysis for subcutaneous fat layer reduction,i± Lasers in 228 Surgery and Medicine, vol.41, no. 10, pp.703.708,2009.
5. Stewart KJ, Stewart DA, Coghlan B, Harrison DH, Jones BM, Waterhouse N. Complications of 278 consecutive abdominoplasties. J Plast Reconstr Aesthet Surg 2006; 231 59(11):1152.1155.
6. Epstein EH Jr, Oren ME. Popsicle panniculitis. N Engl J Med 1970;282:966-7.
7. Karow AM, Jr., Webb WR. Tissue freezing. A theory for injury and survival. Cryobiology 234 1965;2(3):99.108
8. Beacham BE, Cooper PH, Buchanan CS, Weary PE. Equestrian cold panniculitis in women. 236 Arch Dermatol 1980;116(9):1025.1027.
9. Manstein D, Laubach H, Watanabe K, Farinelli W, Zurakowski D, Anderson RR. Selective 238 cryolipolysis: a novel method of non-invasive fat removal. Lasers Surg Med 2008;40:595-239 604.
10. Coleman SR, Sachdeva K, Egbert BM, Preciado J, Allison J.

Clinical Ef.cacy of 241 Noninvasive Cryolipolysis and Its Effects on Peripheral Nerves. Aesthetic plast surg. 2009 242 Jul;33(4):482-488
11. Zelickson B, Egbert BM, Preciado J, Allison J, Springer K, Rhoades RW, Manstein D 244 (2009) Cryolipolysis for noninvasive fat cell destruction: initial results from a pig model. 245 Dermatol Surg 35:1462.147
12. Lee KR. Clinical efficacy of fat reduction on the thigh of Korean women through 247 cryolipolysis. J Obes Weight Loss. 2013;3:1.5.
13. Faul, F., Erdfelder, E., Buchner, A., & Lang, A.-G. (2013). G*Power Version 3.1.7 249

(computer software). Universitat Kiel, Germany.
14. http://www.statcan.gc.ca/pub/82-003-x/2012003/article/11707/c-g/fig1-eng.htm 251
15. Jalian H, Avram MM, Garibyan L, Mihm MC, Anderson R. Paradoxical adipose 252 hyperplasia after cryolipolysis. JAMA Dermatol. 2014;150(3):317-319.
16. Hu F. Measurements of adiposity and body composition. In: Hu FB, editor. Obesity 254 epidemiology. New York: Oxford University Press; 2008. p. 53-83.
17. Meyer PF, da Silva RM, Oliveira G, Tavares MA, Medeiros ML, Andrada CP, Neto LG. 256 Effects of Cryolipolysis on Abdominal Adiposity. Case Rep Dermatol Med. 257 2016;2016:6052194. Epub 2016 Nov 8.
18. Ferraro GA, De Francesco F, Cataldo C, Rossano F, Nicoletti G, Di¯Andrea F. Synergistic 259 effects of cryolipolysis and shock waves for noninvasive body contouring. Aesthetic Plast 260 Surg. 2012;36:666.679.
19. Riopelle JT, Kovach B. Lipid and liver function effects of the cryolipolysis procedure in a 262 study of male love handle reduction. Lasers Surg Med. 2009;82
20.. Shek S, Chan N, Chan H. Non-invasive cryolipolysis for body contouring in Chinese . a 264 .rst commercial experience. Lasers Surg Med 2012; 44: 125.130.
21.. Sun Z, Yang Y, Liu J. Alternative cooling and heating as a novel minimally invasive 266 approach for treating obesity. International journal of Thermal Sciences. 2013;64: 29-39.
22. Kyu Rae Lee, Doyeop Lee, Kidong Ko et al. Clinical efficacy of adipose tissue on abdomen and metabolic variables by Cryo Elsa (Huons, Korea) Obesity Week, (Poster)2017.
